# The road to approved vaccines for respiratory syncytial virus

**DOI:** 10.1038/s41541-023-00734-7

**Published:** 2023-09-25

**Authors:** Tracy J. Ruckwardt

**Affiliations:** Vaccine Research Center, National Institute of Allergy and Infectious Diseases, NIH, Bethesda, MD 20892 USA

**Keywords:** Vaccines, Vaccines

## Abstract

After decades of work, several interventions to prevent severe respiratory syncytial virus (RSV) disease in high-risk infant and older adult populations have finally been approved. There were many setbacks along the road to victory. In this review, I will discuss the impact of RSV on human health and how structure-based vaccine design set the stage for numerous RSV countermeasures to advance through late phase clinical evaluation. While there are still many RSV countermeasures in preclinical and early-stage clinical trials, this review will focus on products yielding long-awaited efficacy results. Finally, I will discuss some challenges and next steps needed to declare a global victory against RSV.

## Introduction

Since the discovery of respiratory syncytial virus (RSV, originally called Chimpanzee Coryza Agent) in 1956, much has been learned about its pathogenesis and the impact of RSV disease in humans. RSV presents a substantial burden in young infants across diverse settings^1,2^. It is the most common cause of acute lower respiratory tract infection (LRTI) and hospitalization in children under 2 years of age^3^. In 2019, it was estimated to cause 33 million cases of acute LRTI, 3.6 million hospitalizations, and over 100,000 deaths in children under 5 years of age^4^. More than 97% of RSV-attributable deaths in children under 5 occur in low- and middle-income countries (LMIC), a high proportion of them occurring in the community rather than in a hospital^4,5^. Most hospitalizations occur in infants less than 6 months old and an estimated 6.6 million acute LRTI infections, 1.4 million hospital admissions, and more than 45,000 RSV-attributable overall deaths occur in this age group globally^4,6^. While most children hospitalized with RSV have no known risk factors, prematurity, chronic lung disease, congenital heart disease and several other factors predispose to severe disease^7,8^. RSV infection in early life has been linked to childhood asthma and impaired lung function, and in a prospective study, avoiding infection in the first year of life substantially lowered the risk of childhood asthma^9,10^.

At the opposite end of the age spectrum, older adults are also at risk for severe RSV disease, particularly the frail elderly or those with comorbid conditions^11–15^. The annual attack rate for older adults generally ranges between 3% to 10%, resulting in an estimate of over 177,000 hospitalization and 14,000 deaths in older adults in the United States every year^14,16^. The burden of RSV in older adults is underestimated because sampling procedures typically used for RSV diagnosis (RT-PCR from nasopharyngeal swabs) have limitations in adults who can have lower viral titers than infected children. Using a wider variety of samples including saliva, serum, and sputum dramatically increases the cases of RSV diagnosed among adults hospitalized with acute respiratory infection^17–20^. Severe RSV disease in older adults has long-term effects, which often include the worsening of prior conditions. Hospital readmission rates are high within 30 days after discharge, and there is substantial health care utilization through 6 months. Patients often require home health services or long-term care facility placement, and there is an increased risk for mortality within the first year^21^.

In summary, RSV exerts a substantial burden on the health of young infants and older adults globally, with impacts that extend well beyond acute infection. Vaccines and other countermeasures that can be used broadly to combat the impact of RSV on these high-risk populations are urgently needed.

### Respiratory syncytial virus

RSV is a member of the *Pneumoviridae* family^22^. It has a negative-sense genome encoding 11 proteins. The nucleoprotein (N), phosphoprotein (P), polymerase (L) and M2–1 transcription processivity factor comprise the ribonucleocapsid, which is encased in an endoskeleton of envelope-associated matrix (M) protein. The M protein lattice coordinates a densely packed viral envelope, studded with the fusion (F), attachment (G), and small hydrophobic (SH) membrane proteins^23–25^. Other RSV proteins include the nonstructural proteins NS1 and NS2, and a regulatory factor translated from a second, overlapping open reading frame in the M2 gene called M2–2. RSV buds from infected cells as filamentous particles but breaks down to asymmetric and spherical particles over time^26^. The G and F glycoproteins are the primary targets of neutralizing antibodies. Despite containing a small central conserved domain, G has the highest genetic diversity, enabling the segregation of viral sequences into two subtypes, A and B, each of which contains multiple genotypes^27^. While one subtype may dominate during a season, A and B subtypes generally cocirculate. F is a type I viral fusion protein, synthesized as single-chain polypeptides that are cleaved by host proteases and displayed as trimers on the viral envelope. RSV F is unusual in that it contains two polybasic cleavage sites, resulting in the release of a 27 amino acid fragment before formation of the mature protein. F is an absolute requirement for viral fusion with the host cell and has a high level of genetic and antigenic conservation^28^. Most protective antibodies target the F protein, and the F-specific antibody palivizumab (Synagis®) has been used to protect high-risk infants from disease since 1998^29,30^.

Despite limited antigenic variability in the most protective antigen, RSV is a seasonal and ubiquitous cause of human disease. RSV infection does not generate durable immunity against reinfection, similar to what is seen for other respiratory viruses^31,32^. Responses to infection are limited, and reinfection is common^33,34^. As seen for other respiratory viruses, changes in human behavior and mitigation efforts after the emergence of severe acute respiratory syndrome coronavirus 2 (SARS-CoV-2) interrupted RSV transmission and disease^35^. RSV attack rates were low in 2020, followed by an atypical outbreak in May of 2021 in the Northern hemisphere or November of 2021 in South Africa^36,37^. By 2022, RSV disease surged a few months earlier than its typical seasonal pattern and joined influenza and SARS-CoV-2 in driving high rates of respiratory disease and hospitalization^38^. The continued impact of RSV on morbidity and mortality makes the development and implementation of effective countermeasures critical, despite disrupted epidemiology and seasonal disease patterns during the pandemic^39^.

### Early RSV vaccines

The RSV vaccine field encountered tragedy shortly after discovery of the virus. Based on technology of the time, a formalin-inactivated (FI) RSV candidate was the first to be tested. Immunity elicited by FI-RSV primed for more severe disease after natural RSV infection – 80% of infected FI-RSV immunized children were hospitalized and two died as a result^40^. Vaccination with FI parainfluenza virus vaccine candidates did not similarly prime for enhanced respiratory disease (ERD) after RSV infection, suggesting a role of RSV-specific immunity. The unexpected failure of FI-RSV made developers balk and approach testing, particularly in antigen-naïve infants, with a high degree of caution. The ERD outcome has been linked to the induction of antibodies with weak neutralizing activity responsible for complement fixation and immune complex deposition and Th2-biased CD4 + T cell responses, a profile that should be carefully avoided when vaccinating antigen-naïve young infants against RSV^41^. There is no precedent for enhanced disease in infants that have had a prior RSV infection, thus live-attenuated vaccines present one of the lowest risks for eliciting unfavorable immune responses in RSV-naïve infants. A major challenge for this approach has been balancing attenuation and immunogenicity^41^.

For several decades, few live attenuated and purified fusion protein or subunit-based vaccine candidates advanced to late-phase testing only to yield unsatisfying efficacy results^41^. While much was learned about RSV biology, epidemiology, and the challenges of protecting the most vulnerable populations, the response to RSV disease has relied primarily on supportive care. Prophylactic monthly administration of palivizumab during the RSV season has proven useful since 1998 to prevent severe disease in a small population of premature and at-risk infants, but it has not demonstrated a therapeutic benefit^42,43^.

### Structure-based vaccine design

Structural determination of the two major conformations of the F protein offered some explanations for the failure of prior vaccines to protect. The structure of postfusion F (postF) was determined in 2011. PostF is highly stable and displays known sites of recognition for neutralizing antibodies including palivizumab and its more potent derivative motavizumab^44,45^. Determining the structure of prefusion F (preF) proved more difficult, and a monoclonal antibody (mAb) was needed to lock the protein in this metastable active conformation^46^. While the F protein undergoes a dramatic and irreversible conformational transformation to postF during fusion, a substantial portion of the protein remains relatively unchanged such that four described antigenic sites (I, II, III, IV) are displayed on both forms of the protein. In addition to these “shared” epitopes, the metastable preF displays conformation-specific antigenic sites referred to as sites Ø and V on the apex^41,46^. Monoclonal antibody discovery efforts to map the antigenic surface of preF have shown that sites Ø and V are targets for the most potently neutralizing antibodies, thus representing major sites of vulnerability^47–49^. Therefore, to elicit the most potent antibodies and in turn, confer the most protection, F-based vaccines need to retain antigenic sites Ø and V. This was the basis for the first prefusion-stabilized subunit vaccine candidate called DS-Cav1^50^. DS-Cav1 was evaluated in a phase 1 clinical trial, providing the first proof-of concept in humans for potent elicitation of neutralizing activity and high potency antibodies targeting the antigenic sites unique to preF^51,52^. Other stabilization solutions followed DS-Cav1, demonstrating superior elicitation of neutralizing activity with a variety of stabilizing mutations^53–55^.

DS-Cav1 activates memory B cells specific for all known antigenic sites, while postF vaccination activates B cells recognizing shared sites I-IV. As a result of eliciting lower potency antibodies, postF vaccination results in a higher fold-increase in binding than neutralization after immunization, and overall lower potency neutralizing activity compared to DS-Cav1^49,56,57^. Thus, the limited immunogenicity and efficacy of many prior vaccines can be linked to the presentation of postF lacking the most critical targets of vulnerability. This is most evident for the “lot 100” FI-RSV vaccine – the preparation method used to produce the vaccine resulted in the absence of preF on the surface, altering the antigenicity away from that of the infectious virus^58^. Several F-based subunit vaccines evaluated were known or revealed to be postF, and elicited immunity could not recapitulate the nature of post-infection human sera, where antibodies specific for preF are responsible for most neutralizing activity^59–63^. An F-based nanomeric micelle vaccine candidate made in Sf9 insect cells was found to display a variety of F conformations and retain an intermediate level of binding to preF only binding antibodies^64,65^. It did not achieve sufficient efficacy for protection from LRTI in late-phase testing in older adults, or for protection of infants of vaccinated mothers in the PREPARE phase 3 trial (NCT02624947). The vaccine elicited an 18.6-fold increase in F-binding IgG, but only a 2- to 3-fold increase in neutralizing activity, a profile like that seen following vaccination with postF antigens^66^.

Determination of the preF structure, and the demonstration that vaccines retaining the preF structure preserved neutralization-sensitive epitopes and elicited supranormal levels of neutralizing activity was a game changer. The field shifted to mAbs targeting preF, and vaccines designed to display the neutralization-sensitive sites Ø and V on the preF apex. The changing landscape for vaccines and mAbs curated by PATH (https://www.path.org/resources/rsv-vaccine-and-mab-snapshot/), and a recent comprehensive review detailing all candidates being tested in humans provide broader perspectives that are not covered by this review^67^.

### Late-phase and approved vaccines

**Fig. 1:**
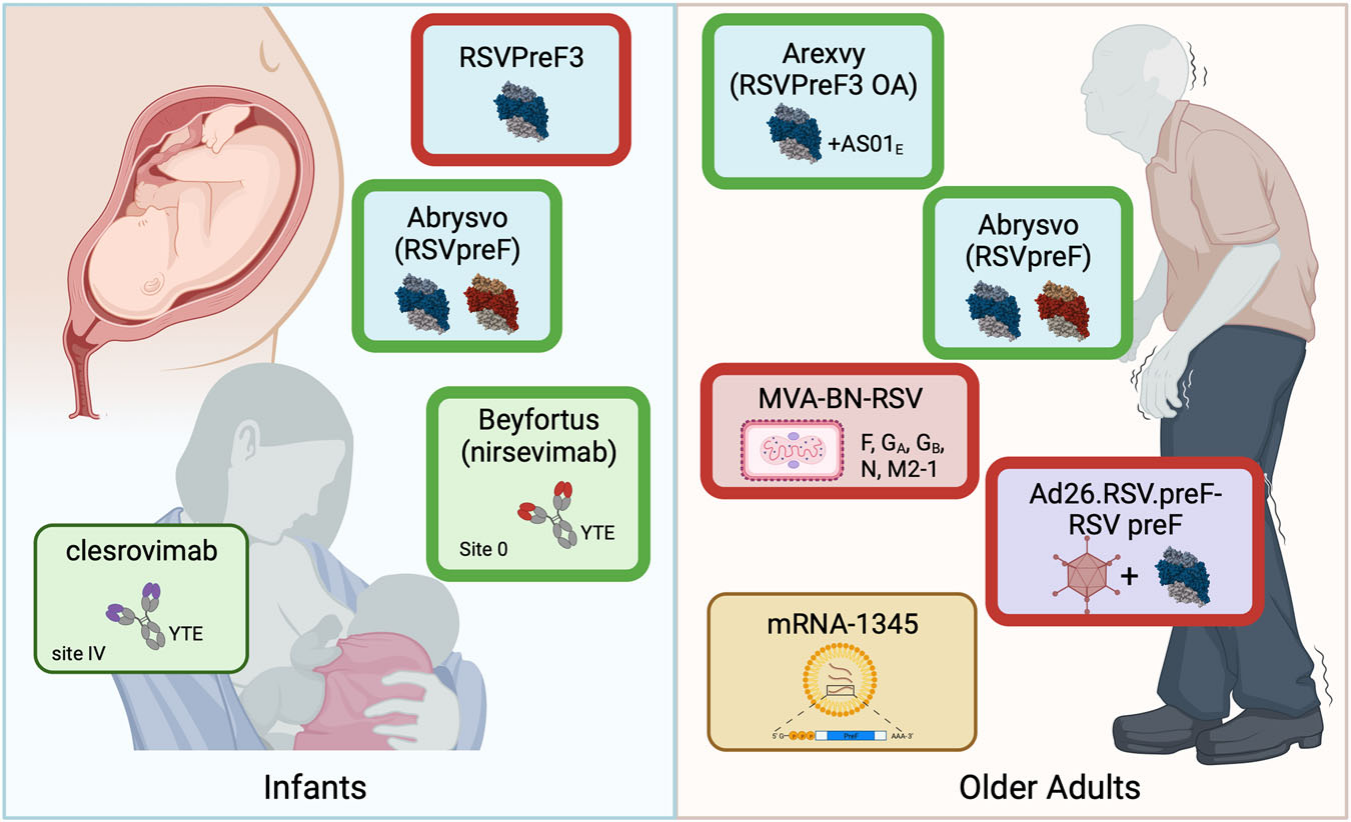
RSV countermeasures completing phase 3 testing in infants and older adults. For protection of infants from RSV disease, the nirsevimab and clesrovimab half-life extended (YTE) mAbs are market-approved or nearing completion of phase 3 evaluation, respectively. The maternal RSVpreF bivalent subunit vaccine Abrysvo has been FDA approved for administration between 32 and 36 weeks of pregnancy, while development of the A subtype RSVPreF3 was stopped due to a safety signal. For protection of older adults, both the AS01_E_-adjuvanted subtype A subunit Arexvy and unadjuvanted bivalent subunit Abrysvo have been approved by the FDA, while development of Ad26.RSV.preF-RSV preF and MVA-BN-RSV vaccines for older adults has been stopped. mRNA-1345 is nearing phase 3 completion. A thick green border indicates market approved, and thick red border indicates that product development and testing has been discontinued. Figure created using biorender.com.

The RSV field is at an unprecedented moment. Late-phase trials of vaccine candidates based on stabilization of the preF protein or leveraging our improved understanding of RSV biology to elicit protective immune responses have delivered or will soon have late-phase efficacy results. Two mAbs and a maternal subunit vaccine are the most advanced candidates for protection in infants, while several vaccine candidates are vying for the older adult market. These most advanced candidates (Fig. 1) are further discussed below.

#### Infant protection from RSV disease

Protection of young infants from severe disease prior to their first exposure can be achieved by bolstering neutralizing antibody responses. One way to achieve this is by direct administration of potent neutralizing antibodies. Currently, the use of the site II-targeting mAb palivizumab is restricted to neonates with extreme prematurity ( < 29 weeks’ gestation) or infants with other selected risk factors due to high cost and the need for monthly administration. It is expected to be replaced by more potent antibodies with extended half-life “YTE” mutations (M252Y/S254T/T256E) in the Fc portion so a single administration can confer protection for an entire RSV season^68^. Increased potency and durability of protection make extending coverage to all infants an achievable goal.

The most advanced mAb, nirsevimab (trade name Beyfortus), binds antigenic site Ø and has >50-fold higher neutralizing activity than palivizumab^69^. Nirsevimab has a comparable safety and side effect profile to palivizumab when compared in palivizumab-eligible infants, and in a phase 2b primary cohort of healthy late-preterm and term infants, a single 50 or 100 mg dose (based on size) prior to the RSV season demonstrated 75% protection against medically attended RSV-associated LRTI^70,71^. It was similarly protective when evaluated in healthy preterm infants^72^. A phase 3 trial (NCT03979313) showed an efficacy of 76% against medically attended LRTI, 77% against hospitalization due to RSV-associated LRTI, and 79% against very severe medically attended LRTI^73^. A pooled analysis demonstrated that prophylaxis with nirsevimab was 80% effective against medically attended LRTI through to 150 days post-enrollment. Based on pharmacokinetic data, the efficacy is expected to be similar in full-term infants as well as those born prematurely or with chronic lung or congenital heart disease^74^. Importantly, despite providing durable protection, nirsevimab does not appear to prevent the development of effective immune responses in infants later infected with RSV^75^. While rare resistance mutations were identified in RSV F protein sequences isolated from infected infants that received nirsevimab, more than 99% of the F protein sequences obtained remained susceptible^76^. Nirsevimab has been market-approved in Europe and the UK and was approved by the Food and Drug Administration (FDA) in July 2023. The Advisory Committee on Immunization Practices (ACIP) has recommended that a dose be given to all infants younger than 8 months entering their first RSV season and a second season dose for high risk groups, and has voted to include nirsevimab in the Vaccines for Children program which provides vaccines at no cost to those who might not be vaccinated because of inability to pay^77^.

Site IV-targeting clesrovimab (MK-1654) is a second half-life extended mAb undergoing phase 3 testing. It recognizes a quaternary epitope, preferentially binding preF over postF and demonstrating high in vivo potency against subtype A and B RSV^78^. Data are available from testing in healthy adults, and modeling studies predict high levels of protection from LRTI through 150 days at a dose of ≥ 75 mg in infants^79–81^. A phase 2b/3 efficacy study in healthy pre- and full-term infants (NCT04767373) is estimated to be completed in 2024, and a phase 3 comparison to palivizumab in high-risk infants (NCT04938830) in 2026.

The alternative approach to protect infants through the first several months of life is maternal vaccination, which relies on the transplacental transfer of neutralizing antibodies. This approach is used successfully for tetanus, influenza, and pertussis, and more recently COVID-19 vaccines^82,83^. Despite not meeting its primary success criteria, the PREPARE trial was the first to demonstrate some short-term efficacy in the offspring of vaccinated women and the feasibility of this approach for RSV F-based vaccines^66,84^. Two preF-stabilized subunit vaccines were next into Phase 3 testing, RSVpreF and RSVPreF3. RSVpreF is bivalent, comprising equal amounts of A and B subtype preF. After testing in healthy adults, it was tested in a phase 2b trial where doses of 120 or 240 µg with or without aluminum hydroxide adjuvant were given to healthy women between 24 and 36 weeks’ gestation^85,86^. Fold-rise in maternal neutralizing antibody in an interim analysis ranged between 11–15-fold for RSV A and 14–18-fold for RSV B. The ratio of RSV-specific antibody transfer through the placenta from mother to infant (ratio of cord blood neutralizing titer to mother’s neutralizing titer) ranged from 1.4 to 2.1 across viral subtypes and vaccine regimens. Observed efficacy point estimates for RSVpreF were 85% against medically attended RSV-associated LRTI and 92% against severe RSV-associated LRTI^86^. In a prespecified interim analysis of a phase 3 trial (NCT04424316), maternal immunization with 120 µg of unadjuvanted RSVpreF (trade name Abrysvo) resulted in vaccine efficacy of 82% against medically attended severe RSV-associated LRTI within 90 days after birth and 69% within 180 days after birth. The estimate for protection from medically attended LRTI was 57% and 51% within 90 and 180 days, respectively^87^. After maternal immunization for prevention of RSV disease in infants under the age of 6 months was recommended by the Vaccines and Related Biological Products Advisory Committee (VRBPAC) in May 2023, the use of Abrysvo between 32 and 36 weeks of pregnancy was FDA approved in August.

The RSV A subtype preF subunit candidate RSVPreF3 was tested at multiple doses in nonpregnant women before further testing at 60 and 120 µg doses during pregnancy^88,89^. Neutralizing activity against RSV A and B rose 13–15-fold and 11–13-fold, respectively, and at-birth antibody transfer ratios were between 1.6 and 1.9. However, due to safety signals in the pivotal phase 3 trial (NCT04605159), enrollment and vaccination in clinical trials evaluating RSVPreF3 were stopped in February 2022^89^.

Given the advancement of both next generation mAbs and maternal vaccines for the protection of young infants, these approaches may complement each other depending on vaccination practices, which may vary greatly by location. The cost of mAbs may limit use in healthy infants in some countries, and it may be difficult to achieve high uptake of maternal antibodies in others. Importantly, both passive antibodies and maternal vaccination have demonstrated a high level of protection in young infants and steer clear of the profile of immunity that was associated with ERD following FI-RSV immunization. These interventions offer hope that protection can be extended beyond only the highest risk infants to protect all infants from both the acute and long-term sequelae of severe RSV disease.

#### Older adult protection from RSV disease

Several vaccines for protection of older adults are completing phase 3 pivotal trials. RSVPreF3 OA, comprising the RSVPreF3 subunit tested for maternal immunization and AS01_E_ adjuvant, was the first RSV vaccine approved by the FDA for prevention of lower respiratory tract disease in adults 60 years of age or older. FDA approval comes after evaluation of RSVpreF3 in young adults (18–40-year-olds) and older adults (60–80-year-olds) at 30, 60, or 120 µg doses with no adjuvant, or with AS01_B_ or AS01_E_ adjuvant. Mean fold-increases in geometric mean titers (GMTs) above baseline for RSV A ranged from 5.5 to 9.6 on day 31 and were durable, ranging from 2.7 to 4.4-fold above baseline at 14 months^90^. A dose effect was observed, and 120 µg of RSVPreF3 with AS01_E_ was selected for further development as RSVPreF3 OA, trade name Arexvy. In a planned interim efficacy assessment of the phase 3 trial (NCT04886596), Arexvy had an overall efficacy of 83% against RSV-related lower respiratory tract disease (LRTD). Efficacy was 94% against severe RSV-related LRTD, and 72% against RSV-related acute respiratory infection. Neutralizing activity increased 10-fold for RSV A and 9-fold for RSV B in the immunogenicity cohort, and high efficacy was observed against LRTD due to both subtypes A and B RSV and across coexisting conditions and frailty status^91^. Arexvy was approved by the FDA for use in adults over 60 on May 3, 2023, making it the world’s first approved RSV vaccine.

Abrysvo, recently approved for prevention of RSV disease in infants of vaccinated mothers, also protects older adults from disease. The bivalent RSVpreF subunit vaccine candidate was tested at multiple doses with and without alum adjuvant in healthy adults between 18 and 49 years of age, eliciting an 11–17-fold (RSV A) and 10 to 20-fold (RSV B) geometric mean fold rise in neutralizing activity across doses and formulations with no benefit for the addition of alum. Titers were maintained 4 to 5-fold above baseline at 12 months post-vaccination^85^. RSVpreF was tested with and without alum at the same doses (60 µg, 120 µg, and 240 µg) alone or with seasonal inactivated influenza vaccine (SIIV). RSV neutralizing titers rose by 7 to 15-fold at one month and remained 3 to 5-fold elevated at 12 months post-vaccination. While RSVpreF was well-tolerated and highly immunogenic with or without SIIV, immune responses to SIIV trended lower when RSVpreF was co-administered^92^. RSVpreF was also tested at doses between 60 and 240 µg in healthy adults between 65 and 85 years old with either alum or CpG/alum. At all doses and formulations, geometric mean titers (GMTs) rose between 5–14-fold against RSV A and B, with a 2–4-fold elevation above baseline at 12 months. CpG/alum did not enhance the response to RSVpreF, and again, responses to SIIV were similar or trended slightly lower when the vaccines were co-administered^93^. Efficacy of Abrysvo (120 µg of unadjuvanted RSVpreF) was 87% against symptomatic RSV in an experimental human challenge study in adults 18 to 50 years of age, concomitant with lower viral shedding in vaccinated than nonvaccinated participants^94^. Finally, in an interim analysis of the phase 3 pivotal trial evaluating Abrysvo in adults over 60 years of age (NCT05035212), efficacy was 67% and 86% against RSV-associated LRTI with at least 2 and at least 3 signs and symptoms, respectively. Efficacy against RSV-associated acute respiratory illness was 62%^95^. Abrysvo was approved by the FDA for prevention of RSV disease in adults over the age of 60 at the end of May 2023. Following FDA approval of both Arexvy and Abrysvo, the ACIP has recommended a single dose of either subunit vaccine for adults over the age of 60 using shared clinical decision-making^96^.

A vaccine candidate using replication-defective adenovirus 26 to encode stabilized preF (Ad26.RSV.preF) resulted in a reduction in RSV infections, viral load, and disease severity when tested in a human challenge model in adults aged 18–50 years old^97^. It was later combined with recombinant preF protein into a single vaccine, Ad26.RSV.preF-RSV preF, which was tested in adults over the age of 65 in a phase 2b trial (NCT03982199). It elicited a 12-fold rise in neutralizing activity against RSV A and 9-fold rise against RSV B fold and had efficacy between 70% and 80% based on clinical case definitions ranging from mild to severe^98^. Ad26.RSV.preF-RSV preF has been under evaluation in a phase 3 trial since 2021 (NCT04908683), but an announcement of discontinuation of the trial and older adult program for the vaccine was made in March 2023.

Two additional vaccines are completing phase 3 pivotal trials for protection in older adults. The COVID-19 pandemic demonstrated that mRNA-lipid nanoparticle (mRNA-LNP) is a viable option to quickly deliver vaccines for infectious diseases at scale^99,100^. Leveraging innovations in mRNA-LNP technology and the superiority of preF immunogens, mRNA-1345 encodes membrane-anchored preF. No peer-reviewed immunogenicity or efficacy data for mRNA-1345 are available, but a press release from the sponsor indicates that efficacy in the ongoing phase 3 pivotal trial in adults aged 60 and older (NCT05127434) is 84% against LRTD with 2 or more symptoms^101^. Breakthrough therapy designation has been granted to mRNA-1345, and the BLA submission to the US FDA is expected to be completed in 2023. The MVA-BN-RSV vaccine candidate uses a nonreplicating modified vaccinia Ankara (MVA) virus to deliver multiple RSV antigens (F, N, M2–1, and G from both A and B subtypes)^102^. Serum neutralizing activity increased less than 2-fold by two weeks after MVA-BN-RSV vaccination, but most vaccinees had an increase in T cell responses to multiple of the five encoded RSV antigens^103^. In a human challenge study, MVA-BN-RSV increased neutralizing activity 2-fold for RSV A and 1.6-fold for RSV B, and point estimates for efficacy ranged between 10% and 89% depending on infection definition^104^. In July of 2023, the phase 3 trial testing MVA-BN-RSV in adults 60 years of age or older (NCT05238025) failed to hit a co-primary endpoint with efficacy estimates against severe LRTD with at least 3 symptoms at 42.9%, and development of the vaccine has been stopped.

Interestingly, most candidates achieving phase 3 efficacy results in older adults elicit at least a 10-fold increase in neutralizing activity and similar durability through one year. As a result, evidence across multiple late-phase trials suggests that this level of immunogenicity confers substantial protection ( ~ 80% or more against severe disease outcomes). Beyond completion of the phase 3 pivotal trials, more work is needed to understand the durability of protection, the need for booster immunizations, and how the vaccines will perform in the highest risk groups, including frail elderly and immunocompromised not represented in clinical trials. Post-marketing phase 4 studies are also needed to resolve concerns about possible safety signals seen in phase 3 trials and ensure a high level of safety as these vaccines are anticipated to be administered to millions of people annually. Further studies are also needed to determine whether responses to vaccines for other indications are affected by coadministration with RSV vaccines such that their clinical effectiveness is reduced. Finally, sharing of any available late-phase data despite the termination of vaccine programs in older adults and infants will be vital for our understanding of protective immunity and to inform future trial design and implementation of vaccines going forward (Fig. 1).

### Remaining challenges

With several effective countermeasures against RSV now approved, we have not yet reached the end of the road. Critical challenges remain around protecting the remaining affected population of children over the age of 6 months and improving equity, education, and surveillance. Meeting these challenges will require contributions from people with many different types of expertise, including policy makers, science educators, and physicians serving high-risk populations. Additionally, as RSV vaccines may soon be distributed widely, we should take the opportunity to assess and ask questions that we may only be able to answer during this transitional time.

While the highest risk populations now have protective interventions, nearly half of RSV-associated hospitalizations and deaths occur in children between 6 months and five years of age^4^. Children over 6 months old are more capable of responding to active vaccination, and improvements in live-attenuated and gene-based vaccines may lead to the elicitation of responses to RSV that safely protect naïve young children^41^. As young and school-aged children are often responsible for transmission to infants and older adults, a vaccine that limits transmission could benefit other target populations. There should be a continued investment in protecting this at-risk, major transmitting population^105,106^. Evaluation of different types of vaccines, particularly if they contain RSV proteins in addition to F, may help reveal the contribution of antibody and T cell responses to other viral antigens as well as other immune mechanisms of protection from disease. This could include antibody effector functions beyond neutralization, and responses that exclusively occur at mucosal sites^31,107^. Based on the FI-RSV experience, it will be critical to avoid eliciting immune responses that could lead to immunopathology following infection, particularly in antigen-naïve infants^108^.

The overwhelming majority of deaths from RSV occur in LMIC, making it imperative that steps are taken to ensure access to interventions in places with the highest burden of disease. Many barriers to deployment in LMIC exist^109^. Low awareness of RSV and limited country-level data are major obstacles to defining the impact that interventions could have. Limited availability and costs of diagnostic testing contribute to the lack of information, which is important for understanding the full burden of disease and benefits of immunization^110,111^. Cost of goods will be another obstacle to preventive approaches in LMIC. Collaborations like the one established between the Bill & Melinda Gates foundation and Pfizer will enable faster and more equitable distribution of a maternal vaccine, and other public-private partnerships or the use of biosimilars may help drive down costs of vaccines less than $5 a dose, the target price for LMIC^109^. Education at the community level is critical in LMIC and globally to raise awareness of the impact of RSV. Increased awareness among health care professionals and communication of the benefits of prevention to high-risk groups prior to the implementation of vaccines will help engender trust and counter the rise in vaccine hesitancy. Efforts to increase patient involvement and communication infrastructure like the RSV Patient Advisory Board should be applauded and expanded^112^.

Widespread surveillance and sequencing efforts are also needed as vaccines and mAbs are implemented. Global viral evolution data, particularly from LMIC, and increased whole-genome data are critical knowledge gaps^27,113^. This is especially important during monotherapy deployment. While it was not intervention driven, natural changes in circulating viruses leading to ineffectiveness of the mAb suptavumab against B subtype RSV is a cautionary tale^114^. So far, sequencing efforts have revealed that nirsevimab escape variants are rare with no increase over time, but nirsevimab escape is possible and there is some natural variability in antigenic site Ø^28,115–117^. While several surveillance and sequencing programs exist, broadening our sequence databases and including whole genome sequences will offer insights into RSV evolution and biology as well as identify any impact of countermeasures. A unified nomenclature will aid such efforts^118,119^.

Finally, the SARS-CoV-2 pandemic shifted RSV epidemiology, bringing challenges but also offering some interesting opportunities^39^. As pneumococcal carriage rates remained relatively stable while viral seasonality was disrupted, one such opportunity was taken to demonstrate that RSV and human metapneumovirus (hMPV) are major contributors to community-acquired alveolar pneumonia, accounting for an estimated 49% and 13% of cases, respectively^120^. Interventions with high efficacy will also alter our decades-long relationship with RSV and have potential to improve overall lung health. This offers opportunities to ask questions about secondary effects of vaccination on long-term disease sequelae and pose questions that may become more difficult or impossible to answer once interventions become standard of care.

### Concluding remarks

Despite tragedy, setbacks, and decades of work toward an RSV vaccine, stabilized preF transformed the field, leading to a crop of promising interventions to significantly reduce RSV-associated morbidity and mortality in high-risk populations. Next-generation monoclonal antibodies offer the possibility of protecting infants through a full season with a single immunization, and several preF-based vaccines boost neutralizing antibody responses by 10-fold or more and confer a high level of protection from severe disease outcomes in both young infants and the elderly. There is much excitement as these long-awaited interventions are approved and being deployed. Further effort should be directed to continued progress on other challenges, and to take advantage of one-time opportunities presented by the upcoming change in our long-standing relationship with RSV.

### Reporting summary

Further information on research design is available in the Nature Research Reporting Summary linked to this article.

### Supplementary information

Reporting Summary
